# Potent Activity of Composite Cyclin Dependent Kinase Inhibition against Hepatocellular Carcinoma

**DOI:** 10.3390/cancers11101433

**Published:** 2019-09-26

**Authors:** Yu-Yun Shao, Yong-Shi Li, Hung-Wei Hsu, Hang Lin, Han-Yu Wang, Rita Robin Wo, Ann-Lii Cheng, Chih-Hung Hsu

**Affiliations:** 1 Graduate Institute of Oncology, National Taiwan University College of Medicine, Taipei 10051, Taiwan; yuyunshao@ntu.edu.tw (Y.-Y.S.); alcheng@ntu.edu.tw (A.-L.C.); 2 National Taiwan University Cancer Center, National Taiwan University College of Medicine, Taipei 10051, Taiwan; 3 Department of Oncology, National Taiwan University Hospital, Taipei 10002, Taiwan; liys6751@gmail.com (Y.-S.L.); s09458017@gmail.com (H.-W.H.); amsirahc77@gmail.com (H.L.); f0078555@gmail.com (H.-Y.W.); ali26262@gmail.com (R.R.W.); 4 Department of Internal Medicine, National Taiwan University College of Medicine, Taipei 10051, Taiwan

**Keywords:** cell cycle, cyclin-dependent kinase, dinaciclib, hepatocellular carcinoma

## Abstract

Alterations in cell cycle regulators are common in hepatocellular carcinoma (HCC). We tested the efficacy of composite inhibition of CDKs 1, 2, 5, and 9 through dinaciclib on HCC. In vitro, dinaciclib exhibited potent antiproliferative activities in HCC cell lines regardless of Rb or c-myc expression levels. Dinaciclib significantly downregulated the phosphorylation of Rb (target of CDKs 1 and 2), ataxia telangiectasia mutated kinase (target of CDK5), and RNA polymerase II (target of CDK9) in the HCC cells. In xenograft studies, mice receiving dinaciclib tolerated the treatment well without significant body weight changes and exhibited a significantly slower tumor growth rate than the mice receiving vehicles. RNA interference (RNAi) of CDKs 1 and 9 was more effective in inhibiting the cell proliferation of HCC cells than RNAi of CDKs 2 and 5. Overexpression of CDK9 significantly reduced the efficacy of dinaciclib in HCC cells, but overexpression of CDK1 did not. In conclusion, composite inhibition of CDKs 1, 2, 5, and 9 through dinaciclib exhibited potent in vitro and in vivo activity against HCC. CDK9 inhibition might be the crucial mechanism.

## 1. Introduction

Treatment of advanced hepatocellular carcinoma (HCC) remains a challenge. Although several antiangiogenic targeted agents and checkpoint inhibitors have been recently approved for the treatment of HCC, the objective tumor response rates of these agents are in the range of 10% to 24% [1,2,3,4,5,6,7,8]. Novel therapy for advanced HCC remains a necessity.

Alterations in cell cycle regulators are common in HCC. Studies have reported that 42% to 73% of human HCCs harbor a specific loss of one allele of RB1 [9,10,11]. Because the protein product of RB1, the retinoblastoma (Rb) protein, controls the entry of S phase, such a loss leads to the activation of cell cycle propagation and cell proliferation. In addition, a loss of functional p16INK4A, an inhibitor for cyclin-dependent kinases (CDKs) 4 and 6, has been identified in up to 70% of HCCs [12,13]. Inhibition of CDKs 4 and 6 has shown potential therapeutic effects for HCC cells in preclinical studies [14,15,16].

CDKs constitute a family of protein kinases involved in regulating cell cycle progression and other cell-cycle-independent functions such as transcription, mRNA processing, and nerve cell differentiation [17,18]. For example, CDK5 has been reported to affect the mitogen-activated protein kinase pathway and regulate DNA damage response [19,20]. CDK9 is involved in the phosphorylation of the C-terminal domain of RNA polymerase II and subsequently stimulates transcription elongation [21]. Notably, prior preclinical studies have reported that CDK5 or CDK9 inhibition could exhibit therapeutic activity against HCC [20,22,23]. We hypothesized that the inhibition of CDKs other than CDKs 4 and 6 might also be effective for HCC cells.

Dinaciclib is a potent inhibitor of CDKs 1, 2, 5, and 9; thus, it can theoretically exert its antitumor effect through both cell-cycle-dependent and -independent mechanisms [24]. Preclinical studies have demonstrated the efficacy of dinaciclib in pancreatic cancer, osteosarcoma, and melanoma [25,26,27]. Clinical trials have also revealed its safety and efficacy in patients with breast cancer and leukemia [28,29,30]. We thus used dinaciclib to examine the efficacy of composite inhibition of CDKs other than CDKs 4 and 6 in HCC.

## 2. Results

### 2.1. Dinaciclib Showed Potent In Vitro Activity in HCC Cells

**Figure 1:**
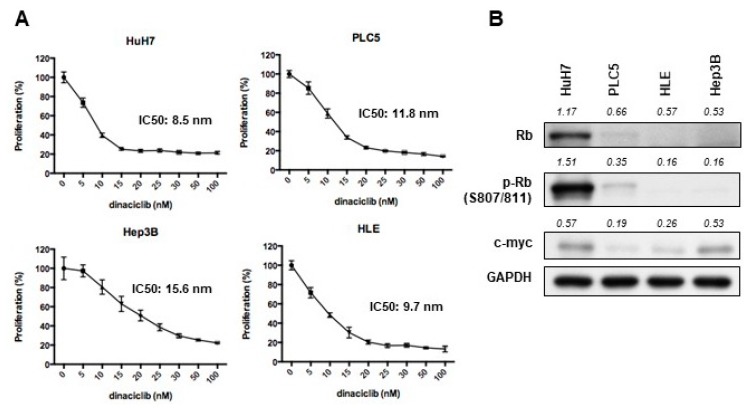

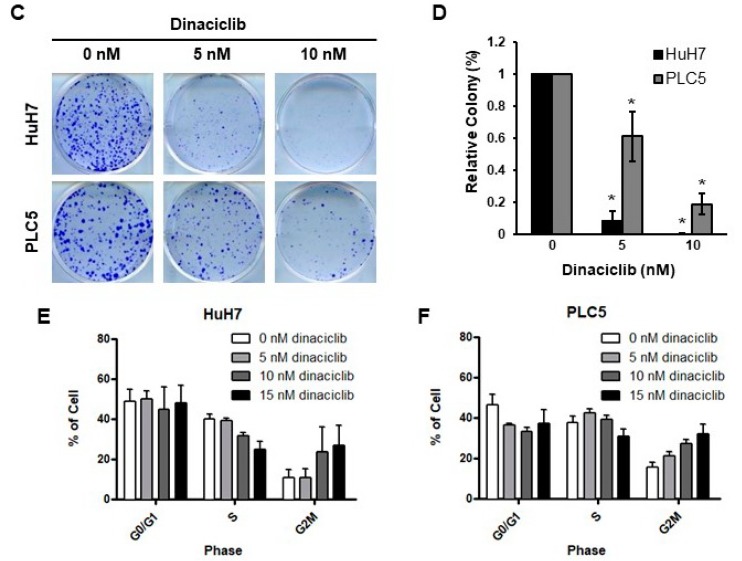
(**A**) MTT assay examining the proliferation of hepatocellular carcinoma (HCC) cell lines after treatment with dinaciclib at the indicated concentration for 72 h. (**B**) Western blot indicating protein expression levels of Rb, phospho-Rb (p-Rb), and c-myc in HCC cell lines. (**C**,**D**) Colony formation assay. HuH7 and PLC5 cells were seeded in 6-well plates and incubated with dinaciclib at the indicated concentration for 10–14 days (* denotes statistical significance compared to treatment with 0 nM dinaciclib). (**E**,**F**) Cell cycle assay. HuH7 (**E**) and PLC5 (**F**) cells were treated with dinaciclib at the indicated concentration for 48 h. After being harvested and stained with PI, the cells were counted and classified according their cell cycle phase using flow cytometry.

Examined using MTT assays, dinaciclib exhibited potent efficacy in reducing cell proliferation in a panel of HCC cell lines, including HuH7, Hep3B, PLC5, and HLE (Figure 1A). The IC50 values ranged from 8.5 to 16 nM. The efficacy did not differ significantly between cell lines with high (HuH7 and PLC5) and low (Hep3B and HLE) Rb expression levels (Figure 1B). The proliferation of cell lines with low c-myc expression, such as PLC5, was also adequately reduced by dinaciclib (Figure 1B). Colony formation assay also demonstrated similar results among HuH7 and PLC5 cells (Figure 1C,D). The cell cycle assay revealed that the HuH7 (Figure 1E) and PLC5 (Figure 1F) cells treated with dinaciclib exhibited cell cycle arrest in the G2/M phase in a dose-dependent manner.

**Figure 2:**
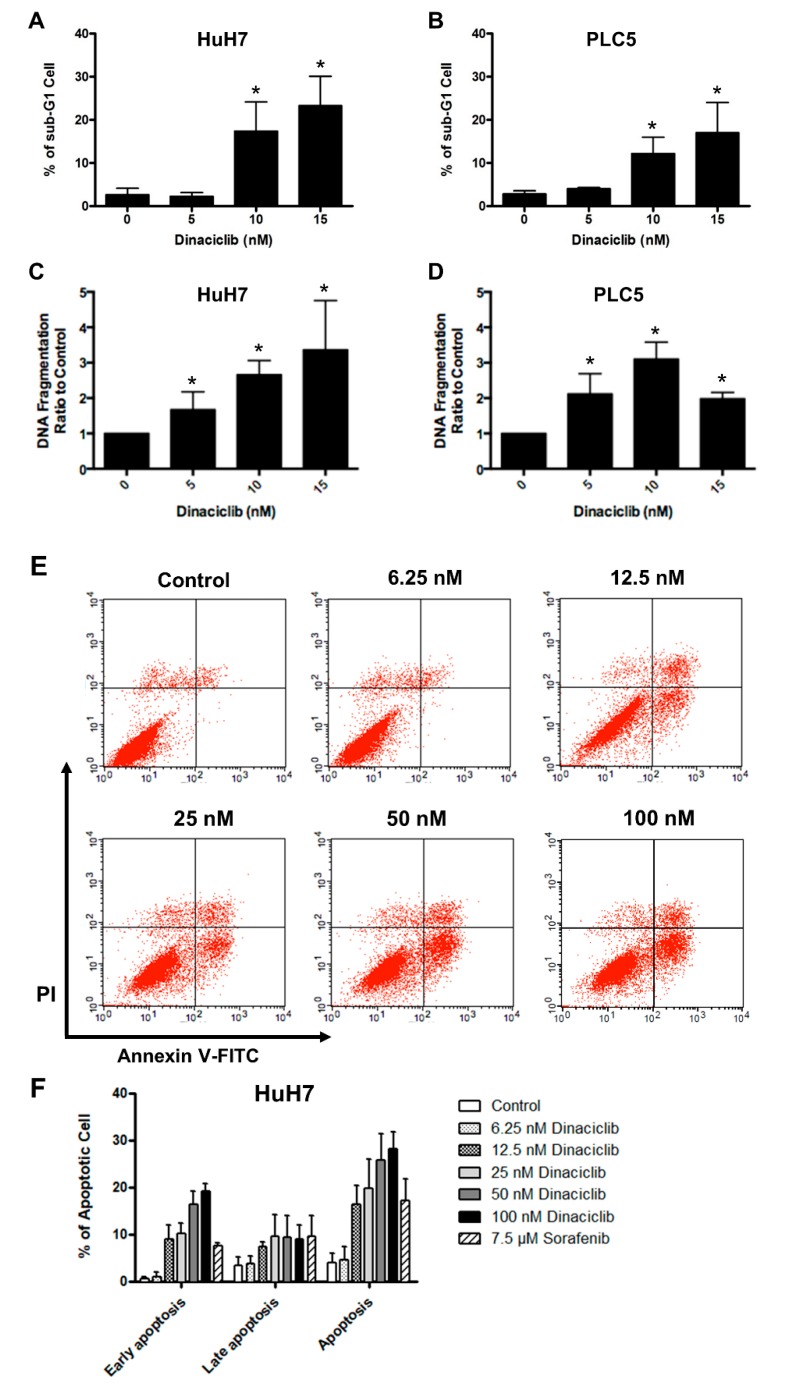
(**A**,**B**) Cell cycle assay indicating the sub-G1 fractions of (**A**) HuH7 and (**B**) PLC5 cells after treatment with dinaciclib for 48 h. (**C**,**D**) Cell death detection ELISA indicating the amount of DNA fragmentation of (**C**) HuH7 and (**D**) PLC5 cells after treatment with dinaciclib for 72 h. (**E**,**F**) Flow cytometry results with HuH7 cells stained with annexin-V and PI after treatment with dinaciclib or sorafenib for 72 h. * denotes statistical significance compared to treatment with 0 nM dinaciclib.

Multiple apoptosis assays demonstrated that dinaciclib treatment induced apoptosis in the HCC cells. After treatment with 10 or 15 nM dinaciclib, the sub-G1 fraction of the HuH7 and PLC5 cells increased (Figure 2A,B). The cell death ELISA also revealed DNA fragmentation in the HuH7 and PLC5 cells after treatment with dinaciclib, even at 5 nM (Figure 2C,D). After staining with annexin-V and PI, we used flow cytometry to determine the number of cells undergoing apoptosis. Dinaciclib dose-dependently induced apoptosis in the HuH7 cells (Figure 2E,F). We found that the number of apoptotic cells engendered by 7.5 μM sorafenib was similar to that engendered by 12.5 nM dinaciclib (Figure 2F).

### 2.2. Dinaciclib Inhibited Function of CDKs 1, 2, 5, and 9 and Reduced Expression of Antiapoptotic Proteins

**Figure 3:**
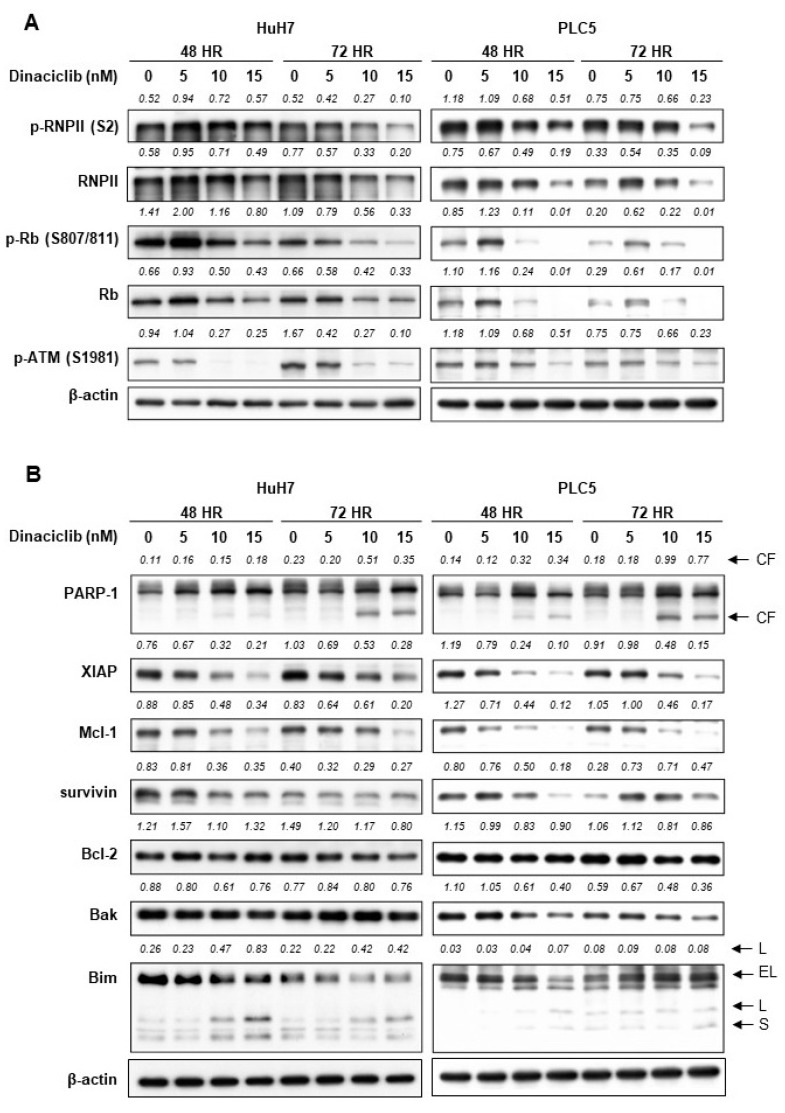
(**A**) Western blots analysis results indicating the expression and phosphorylation of the downstream targets of various CDKs in HuH7 and PLC5 cells treated with dinaciclib. RNPII = RNA polymerase II. (**B**) Western blot analysis results indicating the occurrence of cleaved poly [ADP-ribose] polymerase-1 (PARP-1) and the expression of proapoptotic and antiapoptotic molecules in HuH7 and PLC5 cells treated with dinaciclib.

In both the HuH7 and PLC5 cells, dinaciclib inhibited the phosphorylation of Rb (target of CDK1 and 2), ataxia telangiectasia mutated kinase (ATM) (target of CDK5), and RNA polymerase II (target of CDK9) (Figure 3A).

Dinaciclib induced poly [ADP-ribose] polymerase-1 cleavage in the HuH7 and PLC5 cells (Figure 3B). Dinaciclib reduced the expression of multiple antiapoptotic proteins, including X-linked inhibitor of apoptosis protein (XIAP), induced myeloid leukemia cell differentiation protein (Mcl-1), and survivin, in the HuH7 and PLC5 cells (Figure 3B). Dinaciclib treatment had little effect on the expression levels of B-cell lymphoma 2 (Bcl-2) and Bcl-2 homologous antagonist/killer (Bak).

### 2.3. In Vivo Efficacy of Dinaciclib against HCC

**Figure 4:**
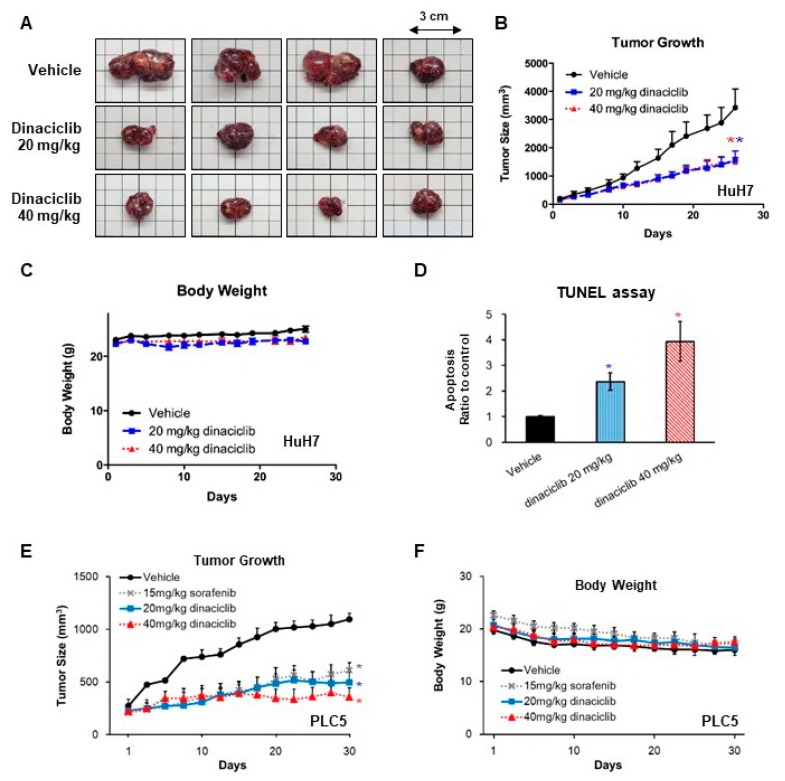
Xenograft studies. (**A**–**D**) Mice were subcutaneously injected with HuH7 cells. Treatment with vehicle (*n* = 6), dinaciclib 20 mg/Kg (*n* = 5), or dinaciclib 40 mg/kg (*n* = 6) was administered 3 times per week. Mice were sacrificed if the tumor size exceeded 1.5 cm in diameter or on the 29th day. (**A**) Representative photographs of tumors harvested from mice. (**B**) Tumor growth curve. * denotes statistical significance compared with treatment with the vehicle alone. (**C**) Body weight of mice. (**D**) TUNEL assay indicating apoptosis in the tumor sections. (**E**,**F**) Mice were subcutaneously injected with PLC5 cells. Treatment with vehicle (*n* = 3), dinaciclib 20 mg/Kg (*n* = 3), or dinaciclib 40 mg/kg (*n* = 3) was administered via intraperitoneal injection 3 times per week. Sorafenib (*n* = 3) was given orally for 5 days every week. (**E**) Tumor growth curve. * denotes statistical significance compared with treatment with the vehicle alone. (**F**) Body weight of mice.

We subcutaneously injected HuH7 cells in mice for xenograft studies. The mice treated with dinaciclib, either 20 or 40 mg/Kg 3 times per week, exhibited significantly slower tumor growth (Figure 4A,B) than the vehicle-treated mice. The tumor growth rates of the mice receiving 20 and 40 mg/Kg were similar (Figure 4B). The mice tolerated dinaciclib well without obvious changes in body weight during the study period (Figure 4C). Using the TUNEL assay, we found that tumors from the mice receiving dinaciclib exhibited a higher number of apoptotic cells than those from the mice receiving the vehicle alone (Figure 4D).

In a similar experiment with PLC5 cells, dinaciclib administered 20 or 40 mg/Kg 3 times per week also exhibited significantly slower tumor growth (Figure 4E) than the vehicle. The tumor inhibition by sorafenib and dinaciclib was similar. Mice tolerated sorafenib and dinaciclib treatment well with stable body weight (Figure 4F).

### 2.4. Key CDKs for the Efficacy of Dinaciclib

**Figure 5:**
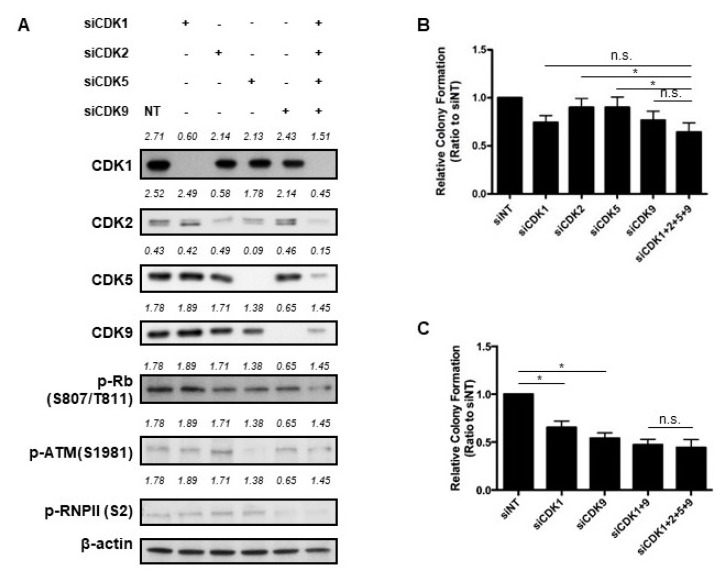

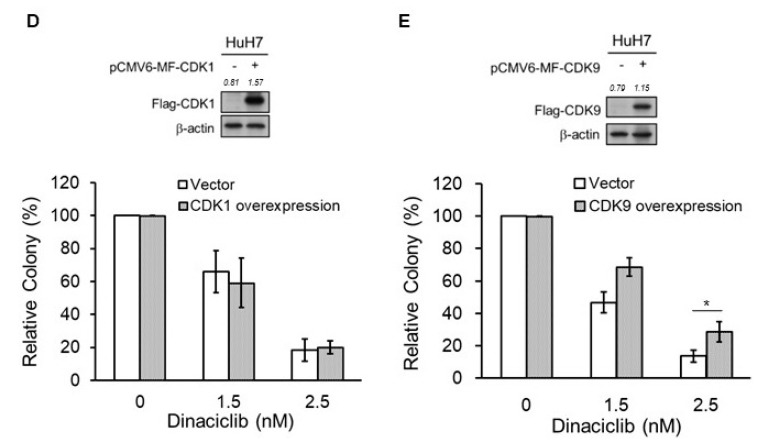
(**A**) Western blot analysis results indicating the expression of various CDKs and their downstream molecules after transfection with siRNAs for 72 h. Nontarget (NT) siRNA served as a negative control. (**B**,**C**) Colony formation assay. After transfection with the indicated siRNAs for 24 h, the cells were seeded and incubated for 14 days. Data are presented as the colony number relative to that of HuH7 cells treated with NT siRNA. * denotes statistical significance of the comparisons between the two manipulations (n.s. = not significant). (**D**,**E**) Colony formation assays. Cells were transfected with the vector overexpressing CDK1 (**D**) or CDK9 (**E**) and then incubated with dinaciclib at the indicated concentration. * denotes statistical significance of the comparisons between the two manipulations.

We used siRNA to inhibit the expression of various CDKs in the HuH7 cells (Figure 5A). Individual knockdowns of CDKs 5 and 9 decreased the level of their downstream molecules, phospho-ATM and phospho-RNA polymerase II, respectively. Individual knockdowns of CDKs 1 or 2 did not result in obvious decrease in phospho-RB levels. When the expression of CDKs 1 and 2 were simultaneously inhibited, phospho-Rb level decreased (Figure S1). Using the colony formation assay, we found that individual knockdowns of CDK1 and CDK9 inhibited colony formation, whereas individual knockdowns of CDK2 and CDK5 did not. Furthermore, simultaneous knockdowns of CDKs 1, 2, 5, and 9 yielded significantly less colony formation than did individual knockdowns of CDK2 and CDK5 (Figure 5B). Although simultaneous knockdowns of CDKs 1, 2, 5, and 9 resulted in less colony formation than did individual knockdowns of CDK1 and CDK9, the difference did not reach statistical significance (Figure 5B). Composite knockdowns of CDKs 1 and 9 and composite knockdowns of CDKs 1, 2, 5, and 9 resulted in similarly reduced colony formation (Figure 5C).

We overexpressed CDKs 1 or 9 in HuH7 cells. Overexpression of CDK1 had minimal effect on the efficacy of dinaciclib (Figure 5D), but overexpression of CDK9 significantly reduced the colony formation inhibition by dinaciclib (Figure 5E).

## 3. Discussion

In this study, we demonstrated that the inhibition of CDKs other than CDKs 4 and 6 was effective on HCC cells. Dinaciclib, an inhibitor of CDKs 1, 2, 5, and 9, was found effective in vitro and in vivo against HCC. Through manipulating the expression of various CDKs, we found that CDKs 1 and 9 may play a bigger role in the efficacy of dinaciclib on HCC cells, and CDK9 might have an even more crucial role because both knockdown and overexpression experiments revealed its importance. Our results support the clinical development of dinaciclib, or other similar CDK inhibitors, as a treatment for HCC [31,32].

Previous studies have demonstrated that CDK9 inhibition was particularly efficacious for HCC treatment with high myc expression because myc may require CDK9 to promote the expression of its downstream targets [22,23]. In the current study, we found that the expression levels of c-myc were not significantly associated with the efficacy of dinaciclib in HCC cells, but CDK9 might be the most crucial CDK among the targets of dinaciclib. The multiple pharmacological targets of dinaciclib, apart from CDK9, could underlie the discrepancy between our observation and those of the previous reports. The other mechanisms of CDK9 inhibition, such as inhibiting Mcl1 expression [32], may also contribute to our findings.

CDK1 inhibition may also contribute to the efficacy of dinaciclib against HCC cells. CDK1, similar to CDKs 4 and 6, is a cell-cycle-dependent CDK. A phase 2 study of palbociclib, an inhibitor of CDKs 4 and 6, revealed its limited efficacy as a single agent for HCC treatment [33]. We previously reported that cyclin E2, a partner of CDKs 1 and 2, was a crucial mechanism for the efficacy of sorafenib against HCC [34], so CDK1 inhibition may have influence on HCC cells distinct from the inhibition of CDKs 4 and 6. Combined inhibition with other CDKs, especially CDK9, may also alter the effect of CDK1 inhibition.

CDK5 has been reported to be a potential target for HCC treatment. Ehrlich et al. demonstrated in xenograft models that Huh7 cells transfected with short hairpin RNA exhibited significantly slower tumor growth than did control cells [20]. Ardelt et al. reported that CDK5 inhibition could potentiate the efficacy of sorafenib. Our results revealed that dinaciclib is a potent inhibitor of CDK5 in addition to CDKs 1, 2, and 9. Our experiments results implied that such composite inhibition of CDKs may be more beneficial than individual CDK inhibition in the treatment of HCC [35].

In our study, dinaciclib exhibited efficacy in cell lines regardless of Rb expression levels, possibly because it inhibits both Rb-associated and Rb-unassociated CDKs. Because clinical HCC samples frequently exhibited various Rb expression, such composite inhibition of both Rb-associated and Rb-unassociated CDKs may help broaden the clinical usefulness of dinaciclib in HCC.

The inhibition of colony formation using combined knockdowns of CDKs 1, 2, 5, and 9 seemed to be less effective compared to that using dinaciclib. Besides the consideration of knockdown efficiency, it can also be attributed to the potential off-target effect of dinaciclib. Dinaciclib could inhibit CDKs 6 and 7, though higher concentration is required. The importance of these other targets mandates future studies.

One of the concerns in the development of CDK inhibitors in the treatment of HCC is bone marrow suppression because many patients with HCC have preexisting cytopenia due to liver cirrhosis and splenomegaly. Although neutropenia was common in patients who received triweekly dosing of dinaciclib [28,36], a weekly schedule of dinaciclib was shown to be more tolerable [30]. In addition, our results revealed that CDKs 1 and 9, especially CDK9, might be the most crucial CDKs in HCC treatment. Future therapeutic agents with better specificity to individual CDKs such as CDK9 may reduce the toxicity and increase the efficacy for HCC patients.

## 4. Materials and Methods

### 4.1. Cell Lines

HCC cell lines, including HuH7, PLC5, and Hep3B, are routinely maintained in our laboratory [37]. The HLE cell lines were purchased from ATCC. The cell lines were maintained in Dulbecco modified Eagle medium with 10% fetal bovine serum, penicillin (100 units/mL), streptomycin (100 μg/mL), L-glutamine (2 mM), and amphotericin B (25 ng/mL). All cell lines were incubated at 37 °C in a humidified incubator with 5% CO_2_.

### 4.2. Cell Proliferation Assays

Cell proliferation was assessed using 3-(4,5-dimethylthiazol-2-yl)-2,5-diphenyltetrazolium bromide (MTT) assay or colony formation assay. For the former, the HCC cells were cultured overnight and treated with dinaciclib (Selleckchem, Houston, TX, USA) at indicated concentrations for 72 h. MTT was added to each well to obtain a final concentration of 0.4 mg/mL. The cells were subsequently incubated at 37 °C for 1.5 h. The reduced MTT dye was dissolved in DMSO, and the absorbance was measured at 540 nm. Cell proliferation was determined by calculating the relative changes in the absorbance. For the colony formation assay, HCC cells were seeded in 6-well plates with a cell density of 300–500 cells per well. The cells were incubated for 14 days. Subsequently, the cells were fixed with 100% methanol and stained with 0.23% crystal violet (Sigma–Aldrich, St. Louis, MO, USA). We then calculated the number of colonies formed.

### 4.3. Western Blot Analysis

All Western blot analyses were performed according to the standard Western blot protocol and the suggestions of the antibody manufacturers. The antibodies specific for CDKs 1, 2, 5, and 9, individually, were purchased from Abcam (Cambridge, UK). The antibodies for α-tubulin, phosphorylated RNA polymerase II, and β-actin were purchased from EMD Millipore (Darmstadt, Germany), Bethyl (Montgomery, TX, USA), and Sigma–Aldrich, respectively. The rest of the antibodies were purchased from Cell Signaling Technology (Beverly, MA, USA).

### 4.4. Cell Cycle Analysis

The cells were treated with dinaciclib at the indicated concentration for 48 or 72 h. After being fixed in 70% ethanol at −20 °C for 24 h, the cells were stained with propidium iodide (PI) at room temperature for 15 min and were later analyzed through flow cytometry to determine the cell cycle phase.

### 4.5. Apoptosis Assay

Sub-G1 fractions of the HCC cells after the indicated treatments were determined according to the methods of the aforementioned cell cycle assay. In addition, according to the manufacturer’s instructions, we used the Cell Death Detection enzyme-linked immunosorbent assay (ELISA) Plus Kit (Roche, Basel, Switzerland) to detect released histones because of DNA fragmentation. Briefly, the HCC cells were treated with dinaciclib for 72 h. The cells were then harvested, lysed, and centrifuged. The supernatant was mixed with the immune-reagent and incubated at room temperature for 2 h. After allowing the mixture to emit signals, we measured its absorbance at 405 nm.

We also used annexin-V and PI staining to detect apoptosis. The cells were treated with dinaciclib, at the indicated concentrations, or with sorafenib (LC Laboratories, Woburn, MA, USA) for 72 h. We then followed the method of the cell cycle assay but stained the treated cells with both annexin-V and PI.

### 4.6. Xenograft Studies

Each BALB/c nude mouse was subcutaneously injected with 1 × 10^6^ HuH7 cells or 2 × 10^6^ PLC5 cells. Treatment was started when the tumor size exceeded 100 mm^3^. The mice were randomized to receive oral sorafenib 15 mg/kg/day for 5 days per week or intraperitoneal injection of vehicle (20% hydroxypropyl-β-cyclodextrin) only, 20 mg/kg dinaciclib, or 40 mg/kg dinaciclib 3 times per week. The tumor size and the mouse weight were measured 3 times per week. The study had ethical approval from National Taiwan University College of Medicine and College of Public Health Institutional Animal Care and Use Committee (Approval No. 20150344).

We used the terminal deoxynucleotidyl transferase (TdT) dUTP nick-end labeling (TUNEL) assay to detect apoptosis in the tumor samples. Tissue sections (thickness, 5 µm), cut from formalin-fixed and paraffin-embedded tissue blocks of tumors, were deparaffinized, rehydrated, and fixed. We then used the DeadEnd Fluorometric TUNEL System (Promega, Madison, WI, USA) and followed the manufacturer’s instructions. Briefly, the fixed sections were treated with solutions including recombinant rTdT at 37 °C for 1 h and stained with PI subsequently. We then counted the cells with high green fluorescence in the nucleus.

### 4.7. RNA Knockdown and Overexpression

We purchased small interfering RNAs (siRNAs) corresponding to CDKs 1, 2, and 5 from GE Dharmacon (Lafayette, CO, USA). The siRNA corresponding to CDK9 and nontarget siRNA were purchased from Thermo Fisher Scientific (Waltham, MA, USA). In total, 1.75 × 10^5^ HuH7 cells were seeded in 6-well plates and transfected with the indicated siRNA (100 nM) using the DharmaFECT 4 Transfection Reagent (Dharmacon) for 24 h.

We purchased vectors overexpressing CDKs 1 and 9 from OriGene (Rockville, MD, USA). Transfection was performed after seeding 2 × 10^5^ HuH7 cells in 6-well plates using Maestrofectin transfection reagent (OmicsBio, New Taipei City, Taiwan) for 48 h.

### 4.8. Statistical Analysis

All statistical analyses were performed using the SAS statistical software (Version 9.4, The SAS Institute, Cary, NC, USA). A 2-sided *p* value of ≤0.05 was considered statistically significant. For continuous variables such as tumor sizes and cell colonies, the independent *t*-test was utilized to compare the results between groups. The Kaplan–Meier method was used to estimate survival, which was compared between groups using the log rank test.

## 5. Conclusions

We demonstrated the in vitro and in vivo activity of composite knockdowns of CDKs 1, 2, 5, and 9 on HCC cells through dinaciclib. CDK9 might be the crucial mechanism. Our data supports the clinical development of dinaciclib or similar CDK inhibitors as the treatment of HCC.

## Supplementary Materials

The following are available online at https://www.mdpi.com/2072-6694/11/10/1433/s1, Figure S1: Western blot analysis results indicating the expression of various CDKs and their downstream molecules after transfection with siRNAs for 72 hours.
