# Supplementary material for: Potent Activity of Composite Cyclin Dependent Kinase Inhibition against Hepatocellular Carcinoma
Source: Cancers (Basel). 2019 Sep 26;11(10):1433. doi: 10.3390/cancers11101433 (PMC6827105; doi:10.3390/cancers11101433)

Figure S1. Western blot analysis results indicating the expression of various CDKs and their downstream molecules after transfection with siRNAs for 72 hours.

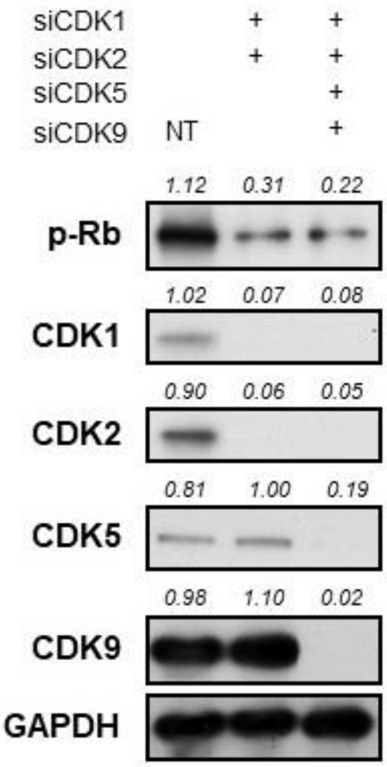

Supplement: Supplementary file 1 [file cancers-11-01433-s001.pdf]
